# Hereditary transthyretin amyloidosis: a case report

**DOI:** 10.1186/s13256-022-03437-0

**Published:** 2022-06-25

**Authors:** Angela Lee, Nowell M. Fine, Vera Bril, Diego Delgado, Christopher Hahn

**Affiliations:** 1 Division of Neurology, Department of Clinical Neurosciences, Cumming School of Medicine, University of Calgary, Calgary, Alberta Canada; 2 Divsion of Cardiology, Department of Cardiac Sciences, Libin Cardiovascular Institute, Cumming School of Medicine, University of Calgary, Calgary, Alberta Canada; 3 Ellen & Martin Prosserman Centre for Neuromuscular Diseases, Toronto General Hospital, University Health Network, University of Toronto, Toronto, Ontario Canada; 4 Division of Cardiology, University Health Network, Toronto, Ontario Canada; 5 Division of Neurology, Department of Clinical Neurosciences, Cumming School of Medicine, University of Calgary, Calgary, Alberta Canada; 6 South Health Campus Hospital, 4448 Front Street SE, Calgary, Alberta T3M 1M4 Canada

**Keywords:** Transthyretin amyloidosis, Neuropathy, Cardiomyopathy, Diagnosis, Case report

## Abstract

**Background:**

Hereditary transthyretin amyloidosis is an uncommon multisystem disorder caused by mutation of the transthyretin protein, leading to peripheral neuropathy often with autonomic features, cardiomyopathy, or a mixed phenotype. Multiple other organ systems can be involved with ophthalmologic, renal, hematologic, gastrointestinal, and/or genitourinary symptoms and signs. This often results in assessments by multiple specialists and significant delays before the diagnosis is recognized. With the recent advent of potentially lifesaving therapies, early diagnosis has become even more important. Our case highlights the protean aspects of this disease as well as the difficulty of making this diagnosis, especially in the absence of a clear family history.

**Case presentation:**

We report the case of a 64-year-old man of East-Asian descent who presented with diarrhea, mild anemia, and symptoms of peripheral neuropathy. Numerous investigations and specialist evaluations did not identify a cause. Progression of neurologic symptoms and the development of new hematologic abnormalities ultimately led to consideration of hereditary transthyretin amyloidosis. The diagnosis was confirmed after re-examining previously acquired gastrointestinal biopsies and pursuing genetic testing, which confirmed a pathogenic mutation in the transthyretin gene. He was subsequently started on a novel gene-silencing therapy. On clinical follow-up 8 months after initiation of therapy, the patient described stabilization of previously progressive numbness, weakness, and weight loss with an unchanged neurologic examination and stable repeat electrophysiologic testing.

**Conclusions:**

Hereditary transthyretin amyloidosis is a challenging disease to recognize in early stages owing to its multisystem and nonspecific manifestations. Recent approval of novel therapies highlights the importance of early diagnosis before irreversible organ damage occurs.

## Introduction

Hereditary transthyretin amyloidosis (hATTR) is an uncommon multisystem genetic disorder caused by deposition of misfolded mutant transthyretin (TTR) protein in multiple organs [1]. Patients typically present with predominantly neurologic and/or cardiac symptoms; however, nonspecific gastrointestinal (GI) symptoms due to autonomic involvement and constitutional symptoms are common. Without treatment, the average life expectancy is 7–11 years from symptom onset [2]. With the approval of effective therapies that can stabilize disease and reduce mortality in hATTR, early identification of affected patients has become increasingly important. We report a case of hATTR that initially presented with predominantly GI and mild neurologic symptoms, leading to significant diagnostic delay.

## Case

A 64-year-old, previously healthy man of East-Asian descent presented to a general internist’s office for evaluation of weight loss and diarrhea. He reported a gradual onset of loose stools over 12 months without blood or change in stool caliber. Preceding the diarrhea by about a year, he described a mild numbness and burning sensation that began in his hands and, after a few months, spread to his feet. There was no weakness either subjectively or on neurological examination.

Fecal immunochemical test within the past year was normal. He has also had a normal screening colonoscopy 6 years prior. Stool testing for ova and parasites was negative. A contrast-enhanced computed tomography scan of the abdomen and pelvis did not show any evidence of neoplasm. He was referred to gastroenterology for evaluation. Esophagogastroduodenoscopy (EGD) and colonoscopy were both unremarkable, including random biopsies. Initial blood work demonstrated mild normocytic anemia (hemoglobin ranging from 127 to 132 g/L, lower limit of normal 137 g/L) and normal C-reactive protein. His neuropathy workup including vitamin B12, hemoglobin A1c, thyroid function, renal function, liver enzymes, and serum/24-hour urine protein electrophoresis (SPEP/UPEP) was normal. Nerve conduction studies done 2 years prior were also normal. The gastroenterologist raised the possibility of choleraic diarrhea, and the patient was started on colestipol with some clinical response.

On clinical follow-up 1 year later, his numbness continued to progress, and he developed weakness in his hands to the point where he could no longer do up buttons or open jars, along with worsening numbness and burning in his feet. He also reported new symptoms of bladder urgency, erectile dysfunction, and orthostatic lightheadedness without syncope. His hematologic parameters had also begun to gradually worsen, and he developed mild leukopenia (total white blood cell count nadir of 3.1 × 10^9^/L, lower limit of normal 4.0 × 10^9^/L) in addition to his anemia. Given his hematologic abnormalities, referral to a hematologist was made for consideration of a bone marrow biopsy and evaluation of cytopenia.

On evaluation by hematology, his symptoms of peripheral and autonomic neuropathy, unexplained GI symptoms, and now hematologic involvement raised suspicion for amyloidosis. His previous duodenal biopsies were re-examined specifically with Congo-red staining and demonstrated amyloid deposition. A bone marrow biopsy with Congo-red staining showed similar findings. Mass spectrometry performed on Congo-red-positive areas of the specimen detected a peptide profile consistent with transthyretin-type amyloid deposition.

**Fig. 1:**
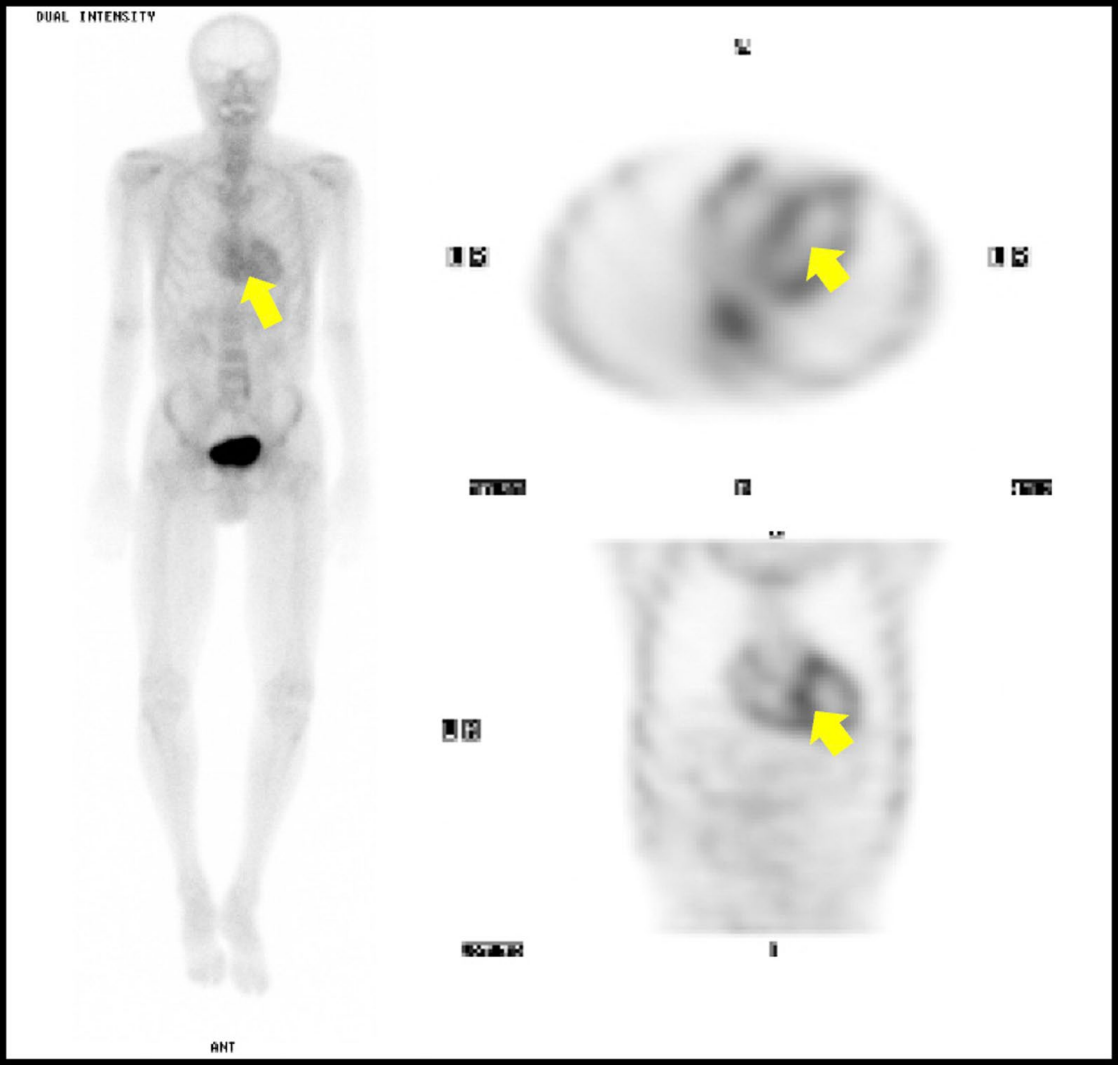
Delayed technetium-99m-pyrophosphate scintigraphy showing increased myocardial uptake (arrows), consistent with cardiac amyloidosis. Of note, there is technetium-99m-pyrophosphate evidence of cardiac amyloid deposition despite absence of overt symptoms of heart failure

SPEP/UPEP with immunofixation as well as serum free light chain assay were unremarkable, making a plasma cell disorder unlikely. On reassessment by a neuromuscular neurologist, examination was notable for atrophy and weakness of intrinsic hands muscles, bilateral foot drop, and sensory loss to pinprick extending to his elbows and knees. Repeat nerve-conduction studies now demonstrated a severe sensorimotor axonal polyneuropathy, consistent with amyloidosis-related neuropathy. Genetic testing confirmed a Val30Met mutation of the TTR gene consistent with hereditary TTR amyloidosis (hATTR). He was then assessed by a cardiologist, and transthoracic echocardiogram demonstrated normal ventricular function with mildly increased left ventricular wall thickness. A technetium-99m-pyrophosphate (PYP) nuclear scintigraphy scan was consistent with cardiac ATTR deposition (Fig. 1), despite the absence of clinical heart failure.

In retrospect, on family history, the patient did report his mother had unspecified heart disease requiring a pacemaker at age 80. She passed away at age 85 from her underlying cardiac disease; however, genetic testing had not previously been done.

**Fig. 2:**
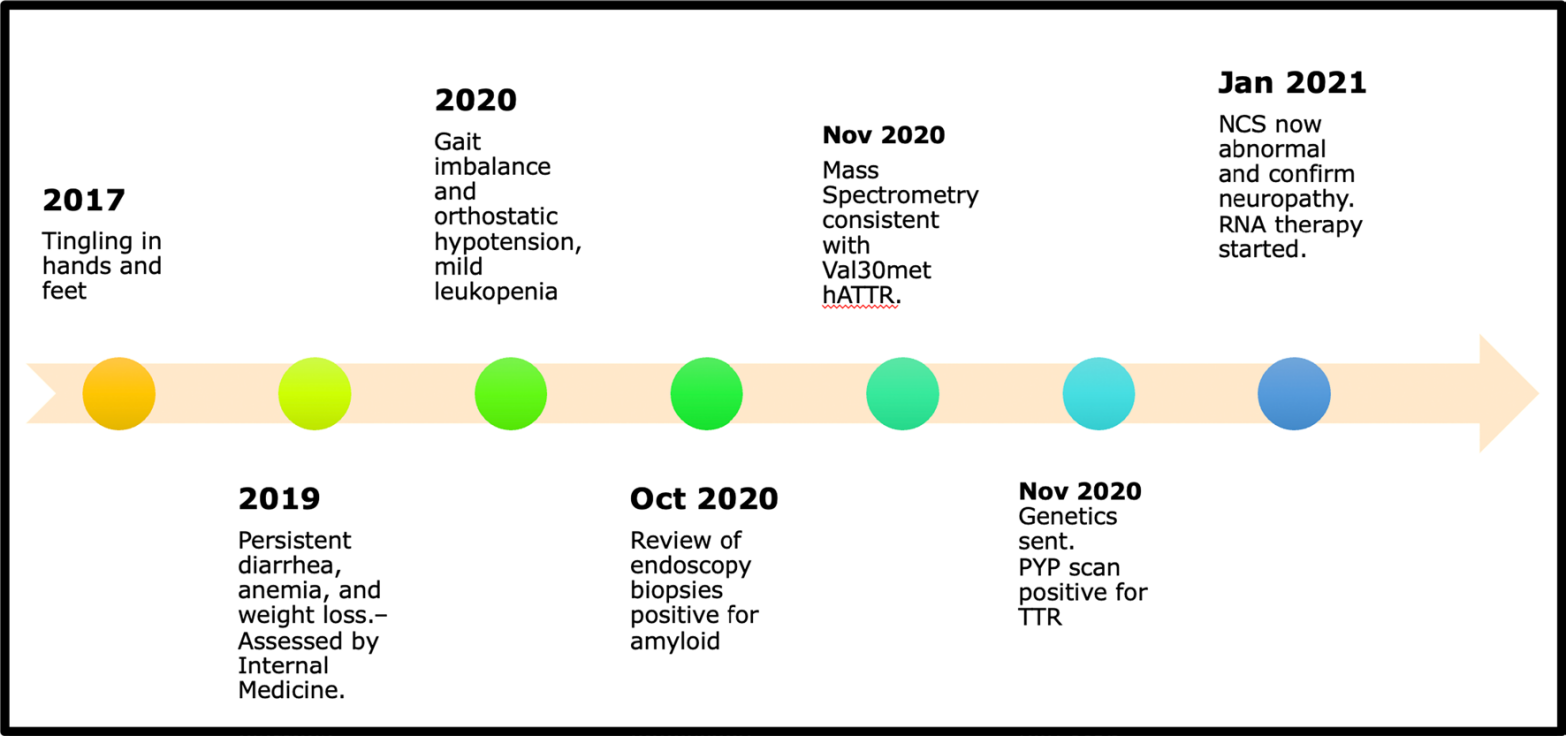
Timeline of patient’s clinical course and investigations. *hATTR* hereditary transthyretin amyloidosis, *PYP* technetium-99m-pyrophosphate, *TTR* transthyretin, *NCS* nerve conduction studies

Given the confirmed diagnosis of hATTR, he was started on patisiran, a novel RNA interference molecule designed to attenuate disease progression by reducing production of mutant TTR. At most recent follow-up, his physical examination demonstrated improvement of his motor strength in his distal hands and legs as well as stable nerve conduction studies 9 months after initiating therapy (Table 1). He has not had any adverse effects related to his treatment and reports subjective stability of symptoms but no improvement. The sequence of events is outlined in Fig. 2.

**Table 1:** Selected nerve conduction study results

Sensory/motor amplitude of nerve studied	Initial study	9-Month follow-up after treatment initiation	Normal value
Median digit 2 SNAP (μV)	3.6	3.8	> 19
Ulnar digit 4 SNAP (μV)	9.0	3.6	> 14
Radial SNAP (μV)	4.8	4.2	> 11
Superficial fibular SNAP (μV)	NR	NR	> 4
Sural SNAP (μV)	NR	NR	> 4
Median APB CMAP (mV)	4.9	6.7	> 5.9
Ulnar ADM CMAP (mV)	4.4	4.7	> 7.9
Peroneal EDB CMAP (mV)	0.2	0.1	> 2.5
Tibial AH CMAP (mV)	0.2	0.1	> 1.1

*SNAP* sensory nerve action potential, *APB* abductor pollicis brevis, *CMAP* compound muscle action potential, *ADM* abductor digiti minimi, *EDB* extensor digitorum brevis, *AH* abductor hallucis brevis

## Discussion

The term amyloidosis encompasses multiple diseases caused by deposition of insoluble protein fibrils in body tissues resulting in end-organ dysfunction. This includes ATTR as well as amyloidosis related to plasma cell disorders (AL or light-chain amyloidosis), products of chronic inflammation (AA amyloidosis as seen with some chronic inflammatory disorders), and organ-specific amyloidosis such as Alzheimer’s disease [1].

ATTR includes genetic forms (hATTR), but also wild-type ATTR (wtATTR). Patients with hATTR typically present with either a neuropathy and/or cardiomyopathy phenotype. Neuropathy is typically sensory, followed by motor involvement as the disease progresses. Neuropathic pain is common. Autonomic neuropathy may manifest with GI symptoms, such as chronic diarrhea or constipation, bladder urgency, orthostatic hypotension, early satiety, and sexual dysfunction. Cardiac symptoms may include heart failure or dysrhythmia, and the classic phenotype is heart failure with preserved ejection fraction. TTR may also deposit in the kidneys or the eyes. Renal deposition may lead to progressive renal failure or nephrotic syndrome, and ocular deposition may lead to vitreous opacities or glaucoma [3]. A mild normocytic anemia is not uncommon [4]. In contrast, wtATTR commonly causes cardiomyopathy in older patients and may include neurologic involvement in the form of carpal tunnel syndrome and spinal stenosis [5, 6]. Neuropathy, when present in wtATTR, is typically mild [4].

Globally, hATTR is a rare disease, but is endemic in certain regions such as Portugal, Sweden, and Japan [5]; thus, a high index of suspicion should be maintained in adult patients with compatible symptoms and ancestry. Recently, genetic testing has become the diagnostic test of choice for patients presenting with neuropathy, given the low sensitivity of many sites of tissue biopsy, although tissue confirmation with mass spectrometry may be helpful in certain settings [5, 7]. Additionally, the PYP scan is a relatively novel tool that has a high sensitivity for cardiac ATTR, although it does not differentiate between hATTR and wtATTR [3].

This case highlights multiple challenges that may delay diagnosis of hATTR. The initial symptoms are often nonspecific, neuropathy can be mild, and family history can be absent or misleading. Nonetheless, early identification remains important, as therapies have been recently approved for patients with either predominant cardiac involvement (tafamidis) [8] or neurologic involvement (inotersen, patisiran) [9, 10]. Time to diagnosis is also important, as the long-term extension studies of these therapies have demonstrated that patients treated earlier in the disease course demonstrated better survival [11] and greater benefit on multiple other measures, including quality of life and disease stability [12, 13].

A multidisciplinary approach is essential both to manage symptoms and to help rule out other conditions. Recent guidelines outline the importance of this approach, with early referral to cardiology and neurology being strongly recommended along with involvement from hematology, when appropriate, to rule out plasma cell disorders [5, 7].

## Conclusions

We describe a case of hATTR associated with Val30Met mutation, initially presenting with prominent GI symptoms, normocytic anemia, and mild neuropathy. Time to diagnosis was 20 months from time of presentation. Challenges to early diagnosis include nonspecificity of symptoms, with many possible and more common etiologies responsible for the patient’s presenting symptoms.

Without treatment, hATTR is a uniformly fatal disease. This case emphasizes the importance of having a low index of suspicion in considering a diagnosis of hATTR early in patients who present with any combination of neuropathy, cardiomyopathy symptoms, and unexplained GI symptoms, particularly in the context of positive family history. Making a timely diagnosis of hATTR may prevent irreversible end-organ damage.

## Data Availability

Data sharing not applicable to this article as no datasets were generated or analyzed during the current study.

## Abbreviations

hATTR: Hereditary transthyretin amyloidosis
TTR: Transthyretin
GI: Gastrointestinal
EGD: Esophagogastroduodenoscopy
PYP: Technetium-99m-pyrophosphate
wtATTR: Wild-type transthyretin amyloidosis
